# Body odour disgust sensitivity is associated with xenophobia: evidence from nine countries across five continents

**DOI:** 10.1098/rsos.221407

**Published:** 2023-04-12

**Authors:** Marta Z. Zakrzewska, Sandra Challma, Torun Lindholm, Sebastian Cancino-Montecinos, Jonas K. Olofsson, Marco Tullio Liuzza

**Affiliations:** ^1^ Department of Clinical Neuroscience, Karolinska Institutet, 17 177 Solna, Sweden; ^2^ Department of Psychology, Stockholm University, 114 19 Stockholm, Sweden; ^3^ Swedish Defence Research Agency, 164 90 Stockholm, Sweden; ^4^ Department of Surgical and Medical Sciences, ‘Magna Graecia’ University of Catanzaro, 88100 Catanzaro, Italy

**Keywords:** olfaction, disgust, prejudice, xenophobia, body odour disgust sensitivity, disease avoidance

## Abstract

Body odour disgust sensitivity (BODS) reflects a behavioural disposition to avoid pathogens, and it may also involve social attitudes. Among participants in the USA, high levels of BODS were associated with stronger xenophobia towards a fictitious refugee group. To test the generalizability of this finding, we analysed data from nine countries across five continents (*N* = 6836). Using structural equation modelling, we found support for our pre-registered hypotheses: higher BODS levels were associated with more xenophobic attitudes; this relationship was partially explained by perceived dissimilarities of the refugees' norms regarding hygiene and food preparation, and general attitudes toward immigration. Our results support a theoretical notion of how pathogen avoidance is associated with social attitudes: ‘traditional norms’ often involve behaviours that limit inter-group contact, social mobility and situations that might lead to pathogen exposure. Our results also indicate that the positive relationship between BODS and xenophobia is robust across cultures.

## 1. Introduction

Disease avoidance is paramount for health and survival, and disease-avoidant behaviours are commonly observed. People adeptly steer away from spoiled foods, bodily wastes and other people who show signs of carrying infectious diseases. When a pathogen threat is perceived, a protective behaviour system (behavioural immune system (BIS); [1]), considered supplementary to our biological immune system, reduces contact with the presumed pathogens [2,3]. Disease avoidance is thought to involve (i) detection of cues of disease that (ii) activate appropriate affective (e.g. disgust) and cognitive (e.g. thoughts of contamination) responses, that in turn (iii) trigger the relevant avoidance behaviour [3]. Individuals vary in their level of disease avoidance, reflecting a trade-off between different fitness-relevant goals (e.g. avoiding unfamiliar foods may reduce the risk of disease, but may have negative effects on nutrition; [4]).

The chemical senses are arguably the most fundamental ways by which organisms detect disease threats. Across many species, conspecifics detect and thwart pathogens using olfaction, resulting in social distancing [5]. Olfaction is a main trigger of disgust and disease avoidance behaviours [6,7]. Disgust is considered a defence mechanism to protect the body from contamination of harmful substances by detecting disease cues [8]. Smells, especially body odours, effectively communicate disease cues [9,10]. The body odour disgust scale [11] assesses the level of reported disgust in response to scenarios involving strong body-related odours that often provide pathogen cues (e.g. urine, sweat and breath; [10]). Scores on the body odour disgust sensitivity (BODS) are positively associated with disgust ratings evoked by sweat bio-samples [12], perceived vulnerability to disease and other forms of disgust sensitivity [11].

Disease avoidance might shape social and ideological attitudes [1]. Individual proneness to interpreting cues as disease-related has been linked to negative attitudes towards specific groups that are perceived as different, e.g. distinct ethnic and sexual minorities [13,14]. Similarly, one's sensitivity to feel disgust has been associated with general attitudes towards others, such as ethnocentrism and political conservatism [15]. Research concerning BODS has shown clear associations between body odour disgust and social attitudes: higher BODS levels are related to more explicit [16] and implicit [17] prejudice against outgroups.

We propose that the effects of individual variability in disgust sensitivity/disease avoidance may manifest in attitudes on two levels: on a target-specific level, towards specific groups or individuals who trigger disease avoidance mechanisms (e.g. ethnic minorities), and on a general level, towards outgroups in general (social conservatism, attitudes towards immigration). This theoretical framework is supported by recent findings from Zakrzewska *et al*. [16], even if the authors do not present it in this way. Namely, a positive association between BODS and negative attitudes towards a fictitious refugee group from Eastern Africa was partially explained by two mediators: general attitudes towards immigration (general level) and how this group was perceived as culturally different in terms of hygiene, sanitary and food practices (target-specific level, the Drashneans).

In the present study, we investigated this association between BODS and xenophobia and its generalizability. Previous findings come from a USA-based sample, a country experiencing an unprecedented level of polarization [18]. If BODS is indeed a manifestation of disease avoidance mechanisms, rooted in evolutionary processes, the observed pattern of relationships should be similar in other samples across the globe. Universal pattern of results would highly benefit the disease avoidance and BIS frameworks, while stark differences between countries could possibly undermine them. In the current study, we tested whether this association would generalize in a large cross-cultural sample, spanning nine countries on five continents. Furthermore, besides using an Eastern African (EA) refugee group in our scenario, we also included an Eastern European (EE) refugee group,^1^ to better understand the role of perceived cultural similarity in the BODS–xenophobia relationship.

Besides providing a global test of generalizability for the BODS–xenophobia association, our research design allowed us to evaluate two theoretical explanations: the *outgroup avoidance account* and the *traditional norms account* (using the terminology from [19]). According to the *outgroup avoidance account,* humans build up resistance to pathogens that are present locally in their environment, and outgroup avoidance is thus a simple protective heuristic. However, the *traditional norms account* builds on recent findings showing that negative attitudes towards immigrants were only related to disgust sensitivity if the immigrants were thought to not adhere to social norms ([19]; see also [20,21]). Social norms serve a key protective role against disease, since they evolve, at least partially, in response to pathogen threats in the environment [22] and affect food preparations [23] or sanitary and hygiene practices [24]. In line with this reasoning, whether a group is perceived as different in terms of such norms (and potentially unwilling to change) can have an influence on attitudes towards this specific group. Perception of outgroups as pathogen threats has been linked to the emotion of disgust (e.g [25]) and thus it seems reasonable to assume that different levels of individual disgust sensitivity might be linked to higher or lower propensity to perceive group norms as different, and thus threatening.

Our hypotheses incorporate the traditional norms account of prejudice, and the two levels (general and specific) at which disease avoidance can affect attitudes towards others. We hypothesized the following:

Pre-registered primary hypotheses:

1. BODS will be related to negative attitudes towards a fictitious outgroup (the Drashnean refugees).
2. Perception of dissimilar food/hygiene/sanitary practices will mediate the relationship between the BODS and negative attitudes towards the Drashneans.
3. Negative attitudes towards the Drashneans will correlate with general attitudes towards immigration.
4. BODS will be related to general negative attitudes towards immigration.
5. Perceived dissimilarity will be related to general negative attitudes towards immigration.
6. We hypothesize that perceived dissimilarity will mediate the relationship between BODS and negative attitudes towards the Drashneans, even when taking into account the shared variance between perceived dissimilarity and general negative attitudes towards immigration.

Pre-registered secondary hypotheses:

7. EA Drashneans will be perceived as more dissimilar in these practices compared with EE Drashneans.
8. Attitudes will be more negative towards the EA Drashneans compared with EE Drashneans.
9. Perceived dissimilarity will mediate the differences in the attitudes towards the two groups.

Lastly, although not of major importance for the current paper, the study was performed during a salient pathogen threat situation (COVID-19 pandemic). We cannot directly compare pre-pandemic results with those in this study, yet according to the relevant theoretical accounts, associations observed before the pandemic should also be present during the COVID-19 pandemic, and the current study investigated whether or not this was the case.

## 2. Materials and methods

### 2.1. Pre-registration

Planned sample size, materials, hypotheses and analysis plan for both waves of data collection were pre-registered on the Open Science Framework Repository (OSF; wave 1, wave 2) as a part of a broader project. Deviations from the plan are marked in the text. As data from both waves were eventually combined into one analysis (see §2.4 *Data analysis*), we followed the more detailed and adequate analysis plan from the second wave pre-registration. Importantly, although the two data collections were pre-registered separately, they follow the same template and share the same theoretical and analytical approach.

### 2.2. Participants

The data were collected using Qualtrics survey software (Qualtrics, Provo, UT). The first data collection was conducted in Sweden and Italy during the first wave of the COVID-19 pandemic in April 2020. The second data collection took place in December 2020 and January 2021 in Canada, Chile, Hong Kong, Kenya, Nigeria, Mexico, New Zealand and the United Kingdom. We aimed to gather data from as many continents as possible for generalization purposes, but for practical reasons, we also wanted to limit the number of translations, and to use the same survey tool in all countries. Our selection of countries was established through discussions with Qualtrics, based on the countries from which they could provide rapid collection of a large sample of responders. In all countries, we recruited participants who were above 18 and below 70 years old. The sample size enabled reliable estimates [26]: it ensured nine observations per parameter, a value that nears the upper recommended level ([27]; upper level = 10). The planned sample size was decreased from 1000 per country in the first wave to 600 per country in the second wave, in order to be able to collect data from more countries, including countries where survey panel participants are fewer, and thus increase the external validity of our results. Participants were randomly assigned to one of the two conditions (Drashnean origin), thus we planned for *n* = 500 (first wave) and *n* = 300 (second wave) in each condition. As the initial Swedish sample turned out to be skewed in terms of age (disproportionately higher numbers of older participants), we collected additional 400 participants in a narrower age range (18–44) to get a demographically representative Swedish sample. Similarly, we recruited additional 70 participants (age range 18–24) from Kenya, where sample's age was also skewed. These additional data were collected after basic screenings, but before any analyses were conducted. The complete dataset was collected prior to the SARS-CoV-2 vaccine becoming available to the public. Participants were granted monetary compensation (€4.0–5.5) according to standard compensation rates in Qualtrics panels in each country. Table 1 contains information about the final sample size and demographics.

**Table 1.** Demographic information about the sample. Gender: F—female, M—male, O—other. Age: mean (s.d.). Education levels show the percentage of participants who chose each option as the highest completed education level: 1—no education; 2—elementary school; 3—high school; 4—college (post-secondary studies); 5—second level (Master's degree); 6—doctoral degree. Note: Mexico is not included in this table, as we excluded data from this country from analysis (see §3.1 *Registered analysis* and electronic supplementary material for more information).

		UK	NZ	Canada	Nigeria	Kenya	HK	Chile	Italy	Sweden	overall
*n*		608	605	610	609	703	609	618	1032	1442	6836
gender		F: 292	F: 287	F: 298	F: 340	F: 334	F: 280	F: 307	F: 511	F: 620	F: 3269
		M: 312	M: 317	M: 305	M: 269	M: 367	M: 327	M: 308	M: 518	M: 817	M: 3540
		0: 4	0: 1	0: 7	0: 0	0: 2	0: 2	0: 3	0: 3	0: 5	0: 27
age		44.7 (15.3)	45.62 (16.0)	47.46 (15.8)	33.53 (11.4)	35.84 (11.8)	36.56 (11.34)	39.02 (13.2)	43.58 (12.7)	45.94 (15.8)	41.98 (14.7)
born in the country (%)		92	71	81	98	98	72	88	96	89	88
education (%)	1	0.5	1.5	0	0.3	0.1	0.8	0.2	0.1	0.1	0.3
	2	1.0	1.0	1.1	0.5	0.3	0.7	1.1	8.1	8.3	3.5
	3	30.8	28.3	26.1	12.5	10.1	23.2	21.7	53.8	42	30.7
	4	49.5	52.4	61.6	58	74.5	56	62.9	13.7	37	47.9
	5	16.3	14.9	9.5	25.5	12.1	16.7	12.5	20.3	11	15.1
	6	2.0	2.0	1.6	3.3	2.8	2.6	1.6	4.1	1.7	2.4

During April 2020, both Italy and Sweden experienced a rapidly increasing number of deaths and ICU cases from COVID-19 [28,29]; data were thus collected at a point where pandemic fear would be expected to be very high. Similarly, during December 2020 and January 2021, the second pandemic wave caused a high number of deaths and cases worldwide.

### 2.3. Measures

Participants completed a self-paced survey, which took about 10 min, and was translated into five languages. We used Swedish and Italian for the first wave of data collection, and English, Spanish and traditional Chinese for the second wave, to ensure that participants in non-English-speaking countries would complete the survey in their native language (all translations available on OSF).

#### 2.3.1. Demographics

We collected demographic information about the participants including gender, age and completed education, which were used as covariates for theoretical reasons. Additionally, we asked whether they were born in the country where the data collection took place (table 1).

#### 2.3.2. Xenophobia and perceived dissimilarity

We used a scenario from Zakrzewska *et al*. [16] to assess explicit xenophobia. In this scenario, participants were introduced to a fictitious outgroup, the Drashnean refugees, from either EA or EE: ‘Imagine the following scenario: There is a country in Eastern Africa (Eastern Europe) which for the purpose of this study we will refer to as Drashnea, that has been experiencing a great deal of civil unrest in the recent years. As a result of these conditions, many people from this country are trying to leave. A large number of these refugees are seeking to immigrate to your country.’ Participants then rated their attitudes towards one of the randomly assigned group by answering eight questions related to (1) their overall attitudes towards the Drashneans, (2) how much they agree that Drashneans could bring health-related problems and (3) criminality into the country if they were allowed to immigrate, and how much they perceived the Drashneans as similar in terms of (4) food, (5) hygiene and (6) sanitary practices. The participants answered the questions using a 10-point scale (from *Strongly disagree* to *Strongly agree* for questions 1–3, and from *Not at all similar* to *Highly similar* for questions 4–6). We also used a feeling thermometer [29] assessed through a visual analogue scale, ranging from 1 to 100. High numbers indicated favourable attitudes towards the Drashneans, 50—neutral attitudes, and low numbers—more unfavourable attitudes. Questions 1–3 were collapsed into an index of attitudes toward the outgroup members (xenophobia). Questions 4–6 were used to assess the perceived dissimilarity of the outgroup members.

#### 2.3.3. General attitudes towards immigration

We used six questions from Faulkner *et al*. [30] to measure general attitudes toward immigration. These assessed participants' opinion about immigrants moving to their country and (i) introducing new skills and practices to the existing culture and economy, and (ii) integrating in the participants' personal life (e.g. by marrying into their family). Participants gave answers on a scale from 1 (*Strongly disagree*) to 7 (*Strongly agree*), higher scores indicating opposition towards immigration.

#### 2.3.4. Body odour disgust sensitivity

BODS is a 12-item scale which measures disgust sensitivity to body odours [11]. Items refer to six types of body odours (faeces, upper body sweat, feet, urine, gas and breath) appearing both in an internal (e.g. ‘*You are alone at home and notice that your feet smell strongly*’) and external (e.g. ‘*You are sitting next to a stranger and notice that their feet smell strongly*’) contexts. Participants rated the extent to which each scenario elicited disgust on a Likert type of scale ranging from 1 (not disgusting at all) to 5 (extremely disgusting).

#### 2.3.5. Attention check

Survey included an attentional check [31]: participants were presented with a longer text that first mentions free-time activities, next reveals that this is a test of whether the participant is carefully reading the instructions. Participants were asked to ignore all response options and type ‘I have read the instructions' (or ‘OK’ in second wave) in the text box for ‘other’ option.

#### 2.3.6. Other measures

Data were collected as a part of a bigger study: we also collected information about all participants' attitudes on issues related to the COVID-19 pandemic (although some items were modified before the second pandemic wave^2^), their political preferences (first wave only) and other ideological measures (second wave only). All measures were pre-registered and can be found, along with relevant hypotheses at OSF.

### 2.4. Data analysis

#### 2.4.1. Combining first and second wave of data collections

Data were collected first in Sweden and Italy during the spring of 2020 (first wave), and the study was expanded to more countries during the winter of 2020 (second wave). Although the two data collections were done (and pre-registered) separately, the materials and theoretical questions (including hypotheses) were the same. For simplicity, we decided to combine the data and analyse it together, rather than as Study 1 and Study 2. Both pre-registrations follow the same analytical approach, with the difference being that we were able to specify our pre-registration further, and correct a few minor errors, before the second wave.^3^

#### 2.4.2. Pre-registered analysis

##### 2.4.2.1 Main analysis

We performed a structural equation modelling (SEM) analysis of the data using R [32], RStudio [33] and the *lavaan* package [34]. First, we conducted a confirmatory factor analysis (CFA) on all questionnaire measures to assure the scales have good validity (Cronbach's α ≥ 0.6) and unidimensionality (RMSEA ≤ 0.08, SRMR ≤ 0.8, TLI ≥ 0.9, CFI ≥ 0.9,^4^ see electronic supplementary material and the pre-registration for more details). The CFA was based on models used in previous studies [11,16]. To account for the ordinal nature of the BODS data, we used the diagonally weighted least squares robust estimator for this scale, and maximum-likelihood (ML) estimator for the other scales. We assessed measurement invariance of the scales in the datasets for all countries to decide whether the datasets could be merged and analysed together or not. The data met the criteria for at least weak (metric) measurement invariance (CFA details and measurement invariance analysis can be found in the electronic supplementary material). Additionally, because we were interested in comparing the means in the two Drashnean conditions, we also looked at measurement invariance between the data from the two groups to make sure we have at least scalar invariance.

**Figure 1.**
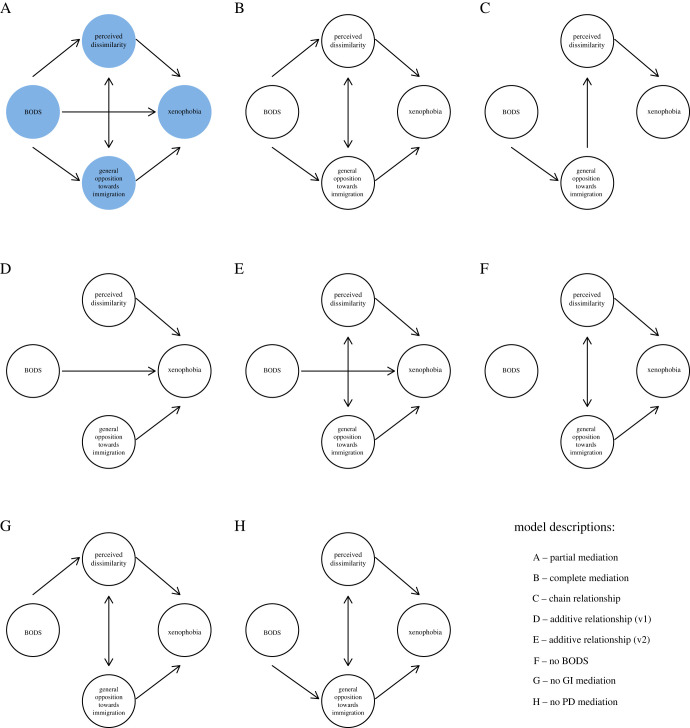
Pre-registered hypotheses and alternative models. Models A–D are the same as those in Zakrzewska *et al*. [16]. Models E–H are additional models pre-registered for this study. Model A (partial mediation) represents our pre-registered hypotheses.

Aside from our hypothesized model, which incorporated the pre-registered primary hypotheses, we looked at the three alternative models previously used in [16] and four additional models (figure 1). This analysis allowed us to answer hypotheses 1 to 6. To decide which model was best we looked at (i) fit indices (change in TLI and CFI > 0.01) (ii) BIC (Bayesian information criterion) and AIC (Akaike information criterion), where smaller values suggest a better model, and (iii) *χ*^2^ difference test and its significance. We used 1000 bootstrap iterations and a ML estimator in the SEM analysis. This analysis allowed us to test hypotheses 1 to 6.

##### 2.4.2.2 Group origin differences

We investigated the effects of our manipulation of group origin (EE versus EA) by assessing a possible interaction effect between BODS and group origin, and an independent effect of the group, by freeing corresponding parameters in the best model from main analysis. First, we assessed measurement invariance in all scales for the two groups and made sure we had scalar invariance. Following, we freed corresponding parameters in the model in order to find a possible interaction effect between BODS and group (EE versus EA) on xenophobia and an independent effect of group on xenophobia. We freed one parameter at a time and kept the changes that improved the model. Starting with the model with all constraints imposed, we freed parameters in this order:

1. xenophobia means,
2. the effect of BODS on xenophobia (direct effect of BODS),
3. perceived dissimilarity means,
4. the effect of BODS on perceived dissimilarity.

We used changes in model fit (CFI and TLI change of greater than 0.01), and the AIC and BIC for guidance in comparisons between subsequent models. In cases where fit indices and information criteria were inconclusive, we used the *χ*^2^ difference test to make the decision (with a more conservative *p*-value threshold, namely *p* < 0.01, due to the large sample). The winning model was used to report differences, if any, between the two fictitious immigrant groups in terms of the four effects stated above. This analysis allowed us to test hypotheses 7 to 9.

Additonally, we decided to free another parameter: the effect of perceived dissimilarity on xenophobia. This was an extra step was added later (not pre-registered) because we thought it would provide information about how similar the entire mediation path (via perceived dissimilarity) is in the two groups. Thus, we also report differences (if any) for the relationship between perceived dissimilarity and xenophobia, the effect of the mediation path via perceived dissimilarity and the total effect of BODS on xenophobia (including also the mediation path via general opposition to immigration).

#### 2.4.3. Exploratory analysis

While we did not have hypotheses about possible cultural differences in the relationship between BODS and xenophobia across different countries, we wanted to see how this relationship looks for each of the nine countries included in the final analysis. To do so, we used a simplified version of the best model from the main analysis (without Drashnean groups) and allowed the coefficients for the total BODS effect to differ across countries. More details about the model are given in electronic supplementary material.

#### 2.4.4. Missing data

We had two cases of missing data in the dataset. Following pre-registration, we removed the data from the two individuals having one case of missing value each.

#### 2.4.5. Data transformations

To ease the modelling, we transformed two variables that had a greater scale than others: we centred age and scaled the feeling thermometer by 10. Thus, feeling thermometer was in the same 1–10 range as other xenophobia items. Reverse-coded items (e.g. the feeling thermometer) were recoded accordingly. Additionally, as we had very few cases of participants indicating their gender as other (*n* = 27), we randomly assigned these participants to either the male or female category.

## 3. Results

Table 2 shows descriptive information about the datasets. In the results section, unless otherwise stated, we report the mean raw estimates with 95% confidence intervals (CI) in square brackets.

**Table 2.** Mean (and s.d.) scores on questionnaires used in the study. EE—Eastern European Drashneans, EA—Eastern African Drashneans. Note: Mexico is not included in this table, as we excluded data from this country from analysis (see §3.1 *Registered analysis* and electronic supplementary material for more information).

		UK	NZ	Canada	Nigeria	Kenya	HK	Chile	Italy	Sweden	overall
BODS		3.6 (0.87)	3.48 (0.88)	3.6 (0.86)	3.95 (0.62)	3.95 (0.64)	3.72 (0.77)	3.86 (0.82)	3.95 (0.71)	3.42 (0.78)	3.71 (0.80)
	external	3.86 (0.89)	3.75 (0.90)	3.87 (0.88)	4.2 (0.68)	4.32 (0.64)	4.01 (0.81)	4.11 (0.84)	4.24 (0.72)	3.87 (0.84)	4.02 (0.82)
	internal	3.35 (0.98)	3.22 (0.99)	3.34 (0.96)	3.69 (0.74)	3.58 (0.86)	3.42 (0.92)	3.6 (0.92)	3.66 (0.84)	2.98 (0.88)	3.39 (0.93)
perceived dissimilarity		5.92 (2.31)	5.96 (2.06)	5.85 (2.07)	6.48 (1.95)	6.04 (2.06)	7.05 (1.9)	6.22 (2.13)	6.65 (2.03)	6.27 (2.22)	6.29 (2.12)
	EE	5.59 (2.25)	5.56 (2.10)	5.53 (2.09)	6.3 (1.98)	6.01 (2.00)	6.75 (1.95)	5.76 (2.01)	6.21 (1.99)	5.82 (2.21)	5.95 (2.11)
	EA	6.25 (2.33)	6.36 (1.94)	6.17 (2.00)	6.65 (1.90)	6.07 (2.12)	7.34 (1.80)	6.67 (2.16)	7.08 (1.98)	6.74 (2.12)	6.63 (2.08)
xenophobia		5.19 (2.19)	5.14 (1.84)	4.7 (1.93)	4.71 (1.76)	4.36 (1.78)	5.6 (1.61)	5.1 (1.89)	5.46 (2.00)	5.49 (2.32)	5.15 (2.03)
	EE	5 (2.21)	5.08 (1.86)	4.79 (1.89)	4.48 (1.71)	4.24 (1.74)	5.52 (1.66)	4.92 (1.84)	5.37 (1.94)	5.42 (2.30)	5.05 (2.01)
	EA	5.37 (2.15)	5.21 (1.82)	4.62 (1.98)	4.95 (1.78)	4.48 (1.82)	5.67 (1.57)	5.28 (1.92)	5.54 (2.06)	5.56 (2.35)	5.24 (2.04)
general opposition towards immigration		3.53 (1.38)	3.38 (1.07)	3.3 (1.12)	3.7 (0.90)	3.55 (0.99)	3.77 (0.83)	3.73 (1.16)	3.93 (1.27)	3.89 (1.40)	3.69 (1.20)

### 3.1. Registered analysis

We had an acceptable level of internal consistency (Crobach's alpha for xenophobia = 0.77, BODS = 0.93, perceived dissimilarity = 0.87, general attitudes towards immigration = 0.76) and goodness of fit for assumptions of unidimensionality for all scales. We achieved at least scalar measurement invariance for all countries in the study except Mexico for all scales. Two of the scales seem to work differently in Mexico: for example, correlations between items and item loadings on the latent variable were deviant. Additionally, there was a Heywood case^5^ when modelling our outcome variable (xenophobia) in the Mexico data. Thus, we decided to exclude data from this country from the main analysis (according to the approach described in pre-registration). We also had to remove one item from the general attitudes towards immigration scale, as it pertained to marriage, which differs across cultures and worked differently across the countries. Details about internal consistency, unidimensionality and measurement invariance are provided in the electronic supplementary material.

#### 3.1.1. Perceived dissimilarity and general attitudes towards immigration partially mediate the relationship between body odour disgust sensitivity and xenophobia

Both mediation models (partial and complete) were better than all other model (TLI and CFI change > 0.01, AIC/BIC change > 1190, *χ*2 differences > 1217, ps < 0.001; table 3). Of the eight models, the mediation models had best-fit indexes, although all fit indices besides SRMR were slightly below optimal (table 3). Although the partial and full mediation models fit the data just as well, information criteria and *χ*^2^ test point to the partial mediation model being better.

**Table 3.** Fit information for the hypothesized and alternative models. $\chi^{2}$—$\chi^{2}$ of the model, d.f.—degrees of freedom; RMSEA—root mean squared error approximation; 95% CI—95% confidence interval for the RMSEA; SRMR—standardized root mean square residual; TLI—Tucker–Lewis fit index; CFI—comparative fit index; AIC—Akaike information criterion difference between hypothesized model (model A) and the indicated model; BIC—Bayesian information criterion difference between hypothesized model (model A) and the indicated model; $\chi_{\mathrm{diff}}^{2}$—$\chi^{2}$ difference between hypothesized model (model A) and the indicated model; *p*—*p*-value for the $\chi^{2}$ test comparing indicated model with hypothesized model (model A). Model A was the reference model for all model comparisons. Models: model A—partial mediation; model B—full mediation; model C—chain, model D—multiple regression v1; model E—multiple regression v2); model F—no BODS; model G—no general immigration attitudes mediation; model H—no perceived dissimilarity mediation.

	$\chi^{2}$	d.f.	SRMR	RMSEA	95% CI	TLI	CFI	AIC	BIC	$\chi_{\mathrm{diff}}^{2}$	*p*
model A	17 695	303	0.063	0.092	[0.090, 0.093]	0.820	0.844	—	—	—	—
model B	17 703	304	0.063	0.092	[0.090, 0.093]	0.821	0.844	6	−1	8	< 0.01
model C	22 119	305	0.099	0.102	[0.101, 0.103]	0.776	0.804	4420	4406	4424	< 0.01
model D	22 846	306	0.114	0.104	[0.103, 0.105]	0.770	0.797	5145	5124	5151	< 0.01
model E	21 186	305	0.084	0.100	[0.099, 0.101]	0.786	0.812	3487	3474	3491	< 0.01
model F	21 223	305	0.087	0.100	[0.099, 0.101]	0.785	0.812	3524	3510	3528	< 0.01
model G	19 801	306	0.084	0.097	[0.095, 0.098]	0.801	0.825	2100	2080	2106	< 0.01
model H	18 912	306	0.083	0.094	[0.093, 0.095]	0.810	0.833	1211	1190	1217	< 0.01

**Figure 2.**
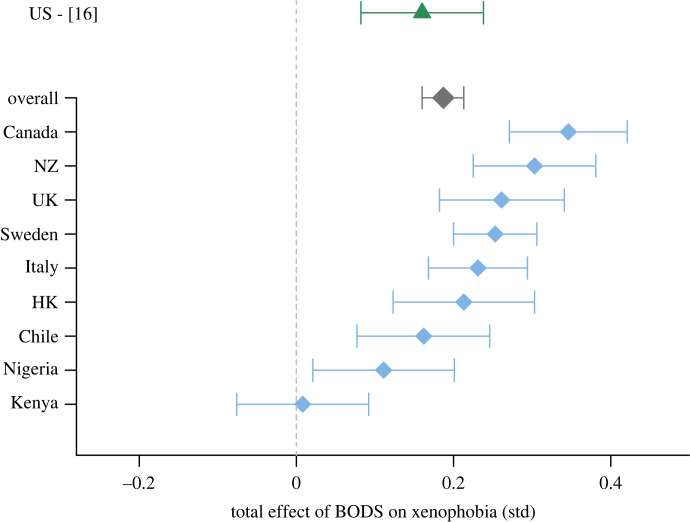
Total effect of BODS on xenophobia in each country. Standardized estimates of total effect of BODS on xenophobia (including the two mediation paths via perceived dissimilarity and general attitudes towards immigration) with corresponding 95% CIs. We also show the estimates for all countries (from the partial mediation model A in the main analysis) and the estimate from the original study on a US sample [16]. NZ—New Zealand, HK—Hongkong, UK—United Kingdom.

**Figure 3.**
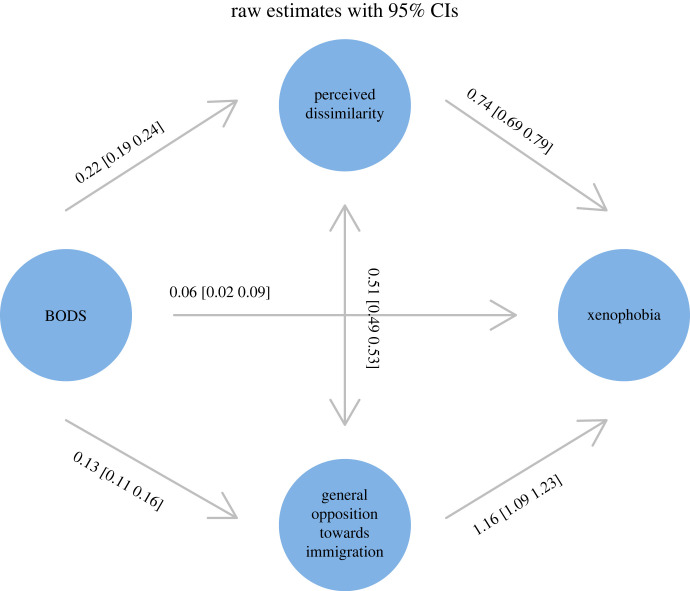
Standardized estimates from the best model (partial mediation), with corresponding 95% CIs. Latent variables are marked with circles.

In the partial mediation model, BODS was positively related to xenophobia (figure 2, ‘overall’), perceived dissimilarity (0.29 [0.27, 0.32]) and general attitudes towards immigration (0.64 [0.6, 0.69]). The total positive effect of BODS was 0.37 [0.32, 0.43] including the mediation path via perceived dissimilarity (0.16 [0.14, 0.18]), the mediation path via general opposition to immigration (0.16 [0.12, 0.19]) and a direct effect (0.06 [0.02, 0.1]). Age was positively (0.01 [0.01, 0.01]) and education negatively (−0.06 [−0.08, −0.04]) related to xenophobia, and men were more xenophobic than women (0.06 [0.02, 0.1]). Standardized estimates of relationships between variables in the winning model are presented on figure 3. Electronic supplementary material, figure S1 shows factor loadings for the latent variables.

#### 3.1.2 Group origin manipulation changed the perceived dissimilarity of the group, but not the prejudice towards the group

**Figure 4.**
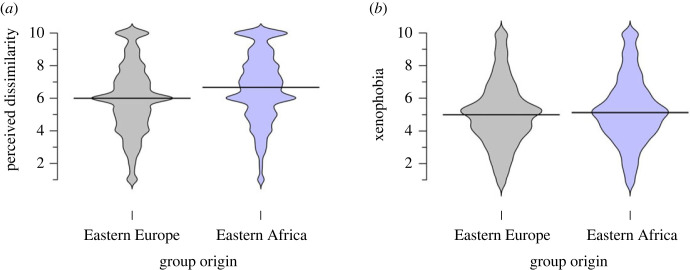
Overall perceived dissimilarity (*a*) and xenophobic attitudes (*b*) towards the fictitious immigrant groups coming from Eastern Europe (grey) or Eastern Africa (blue). The horizontal black lines mark median values, and the width of each plot indicates distributions of scores.

The hypothesized (partial mediation) model outperformed other models; thus, we tested the group effects on this model. The results indicate that group origin manipulation did not change the xenophobic attitudes towards the group, but it did change how dissimilar the group was perceived (figure 4). Namely, EA Drashneans were perceived as more dissimilar than the EE Drashneans (EA: 6.99 [6.92, 7.06], EE: 6.44 [6.37, 6.51], with a difference of 0.55, [0.45, 0.64]). Both groups were on average rated as more dissimilar than similar (ratings greater than 6 on a 1–10 scale). As shown in figure 4, the overall distribution of ratings was similar for the two Drashnean groups.

#### 3.1.3. The positive effect of body odour disgust sensitivity on xenophobia via perceived dissimilarity was similar for both groups

The effect of BODS on perceived dissimilarity was identical in both groups (EE: 0.34, [0.29, 0.39]; EA: 0.34, [0.29, 0.39]; difference of 0 [−0.07, 0.06]). The model allowing for this difference was not better than the more constrained model (according to all three criteria stated in §2.4.2.2 *Group origin analysis*; table 4). Thus, the only model which showed a benefit of including group differences was the model allowing the mean of perceived dissimilarity to vary. In this model, the mediation path via perceived dissimilarity, and the total effect of BODS were set to be the same no matter the Drashnean origin; in other words, there was no interaction. The standardized estimates from this model, and the best model from main analysis (without group effects) are quite consistent for both the mediation via perceived dissimilarity and the total effect of BODS. Table 4 shows comparisons of the models, according to the scenario described in Materials and methods (§2.4.2.2 *Group origin analysis*); table 5 includes the standardized estimates from both models. Electronic supplementary material, figure S2 shows factor loadings for the latent variables.

**Table 4.** Fit information for the models testing group effects. PD—perceived dissimilarity; BODS—body odour disgust sensitivity; $\chi^{2}$—$\chi^{2}$ of the model, d.f.—degrees of freedom; RMSEA—root mean squared error approximation; 95% CI—95% confidence interval for the RMSEA; SRMR—standardized root mean square residual; TLI—Tucker–Lewis fit index; CFI—comparative fit index; AIC—Akaike information criterion difference between the models; BIC—Bayesian information criterion difference between the models; $\chi_{\mathrm{diff}}^{2}$—$\chi^{2}$ difference between the models; *p*—*p*-value for the $\chi^{2}$ test; BODS—body odour disgust sensitivity; PD—perceived dissimilarity. Comparative values are always for the indicated model versus the previous best model (row above). The tilde (∼) sign denotes regression (can be read as: ‘predicted by’).

	$\chi^{2}$	d.f.	SRMR	RMSEA	95% CI	TLI	CFI	AIC	BIC	$\chi_{\mathrm{diff}}^{2}$	*p*
all constraints	19 043	681	0.111	0.089	[0.088, 0.09]	0.831	0.835	—	—	—	—
xenophobia means	19 038	680	0.111	0.089	[0.088, 0.09]	0.831	0.835	−3	4	5	0.03
all constraints	19 043	681	0.111	0.089	[0.088, 0.09]	0.831	0.835	—	—	—	—
xenophobia∼BODS	19 043	680	0.111	0.089	[0.088, 0.09]	0.831	0.835	2	9	0	0.84
all constraints	19 043	681	0.111	0.089	[0.088, 0.09]	0.831	0.835	—	—	—	—
PD means	18 915	680	0.111	0.089	[0.087, 0.09]	0.832	0.836	−126	−119	128	< 0.01
PD means	18 915	680	0.111	0.089	[0.087, 0.09]	0.832	0.836	—	—	—	—
PD∼BODS	18 915	679	0.111	0.089	[0.088, 0.09]	0.832	0.836	2	9	0	0.95
PD means	18 915	680	0.111	0.089	[0.087, 0.09]	0.832	0.836	—	—	—	—
xenophobia∼PD	18 913	679	0.111	0.089	[0.088, 0.09]	0.832	0.836	−1	6	3	0.1

**Table 5.** Standardized estimates from the best model (complete mediation) with and without varying group effects. Standardized estimates and corresponding 95% CI (in brackets). Estimates for the ‘group effects vary’ come from the model where mean perceived dissimilarity was allowed to vary between the two Drashnean groups. BODS—body odour disgust sensitivity; immigration attitudes—general attitudes towards immigration; age—age (centred); EE—Eastern European Drashneans, EA—Eastern African Drashneans; mediation via immigration attitudes—mediation path via general attitudes towards immigration; mediation via perceived dissimilarity—mediation path via perceived dissimilarity. The tilde (∼) sign denotes regression (can be read as: ‘predicted by’).

	partial mediation model	
	group effects fixed	group effects vary
xenophobia∼BODS	0.03 [0.01, 0.05]	0.03 [0.01, 0.05]
xenophobia∼immigration attitudes	0.59 [0.56, 0.61]	0.59 [0.57, 0.61]
xenophobia∼perceived dissimilarity	0.38 [0.36, 0.4]	0.38 [0.36, 0.40]
xenophobia∼gender	0.03 [0.01, 0.04]	0.03 [0.01, 0.04]
xenophobia∼education	−0.05 [−0.06, −0.03]	−0.04 [−0.06, −0.03]
xenophobia∼age	0.14 [0.12, 0.15]	0.13 [0.11, 0.15]
immigration attitudes∼BODS	0.13 [0.11, 0.16]	0.14 [0.11, 0.17]
perceived dissimilarity∼BODS	0.21 [0.19, 0.24]	0.23 [0.21, 0.26]
mediation via immigration attitudes	0.08 [0.06, 0.09]	0.08 [0.07, 0.10]
mediation via perceived dissimilarity	0.08 [0.07, 0.09]	0.09 [0.08, 0.10]
total BODS effect	0.19 [0.16, 0.21]	0.20 [0.17, 0.23]
dissimilarity difference (EA - EE)		0.47 [0.40, 0.54]

### 3.2. Exploratory analysis

Figure 2 shows total BODS effects in each country, obtained using a simplified version of the best model from the main analysis (partial mediation model). Figure 2 suggests that the relationship between BODS and prejudice is strongest in Western cultures, especially in English-speaking countries. However, model comparison did not provide a clear conclusion as to whether allowing for the BODS and xenophobia relationship to differ across countries is warranted or not (electronic supplementary material, table S3). Future research should investigate this exploratory question more thoroughly.

## 4. Discussion

Understanding the link between disease detection/avoidance and xenophobia is critical for understanding the psychology of inter-group processes. Here, we focus on BODS, a potent sensory disease avoidance function, and how it is associated with xenophobic attitudes. Specifically, the present research used a large sample and a pre-registered set of hypotheses to extend previous findings of a positive relationship between BODS and explicit xenophobia [16] across countries and continents. Of particular interest was to understand whether or not body odour disgust is linked to negative attitudes to refugees because of the perceived dissimilarity of the refugees. We found that this was the case, and that our findings generalized well across most countries.

Most importantly, we show that how strongly people report to be disgusted by body odours is related to negative attitudes towards a fictitious refugee group (i.e. effect of BODS on xenophobia) and our overall effect size was very similar to previous findings in participants from the USA [16]. In the current study, data were analysed from nine culturally different and large samples in Africa, North America, South America, Europe, Asia and Oceania. The observed result was partially explained by how respondents perceived the refugee group as different in terms of food, hygiene and sanitary practices, and general attitudes towards immigration. This is in line with the disease avoidance theory that aims to explain suggesting that social behaviours and attitudes are connected to avoiding pathogens. Importantly, our results indicate that the relationship between BODS and xenophobia generalizes across populations, and can be partially explained by perceived outgroup norms. Our results thus provide support for the *traditional norms account* [2,4,19]. Hence, rather than geographical or genetic difference, perceived similarity in food preparation practices seems to be a driver of xenophobic attitudes, and it partially mediates the relationship between other key elements of disease avoidance (i.e. BODS) and xenophobia.

We extended previous findings by comparing attitudes towards the unfamiliar fictitious group from EA to attitudes towards a potentially more similar (at least for western cultures) fictitious outgroup coming from EE. Indeed, the EA Drashneans were consistently rated by most respondents as more dissimilar compared with the EE Drashneans; with the exception of Kenya, which is located in Eastern Africa. This manipulation potentially allows for better causal inferences, even if it worked on the intermediate variable only (perceived dissimilarity) and not the outcome (xenophobia); the difference in perception did not translate to higher levels of xenophobia for the EA Drashneans. This result is at odds with our hypothesis as we expected that the unfamiliar group would be both perceived as more different and elicit more negative attitudes (hypothesis 8 in *Secondary hypotheses*). However, even though EA Drashneans were perceived as more dissimilar, both groups were generally rated as being quite dissimilar, which might explain the lack of differences in the attitudes towards the two groups. Hence, while perceived group similarity is important in understanding the link between BODS and social attitudes, understanding the underlying mechanisms by which dissimilarity operates in these processes needs further exploration.

One limitation of the current study is that it is cross-sectional, comparing attitudes of individuals at one point in time. Exploring changing attitudes in a longitudinal perspective would add important knowledge on how the disgust/xenophobia relation evolves. As with most behavioural research, our study is also vulnerable to sample bias. For example, our study might have over-sampled from the more educated portions within the populations of reference. This might have an impact on the overall levels of xenophobia, since education typically is associated with lower levels of prejudice [35]. Similarily, our sample might be selective in terms of personality factors (e.g. openness to experience), which are also known to relate to prejudice (e.g. [36,37]). However, given the strengths of our study (e.g. the size and demographic stratification of the sample) in combination with the fact that results generalized well across nine countries, we are confident in the generalizability of our findings in a global context.

Our study points to several new lines of investigations relevant for future research in the field. Although explored only superficially in this study, there seems to be certain variability in the strength of the relationship between BODS and social attitudes. The effect was largest in Canada, and several other Western, English-speaking countries, but it was absent in Kenya—one East African country in our study. Such variability is not unexpected, as recent multi-country studies show variation even in highly robust findings [38]. It would be interesting to see if this variability is related to specific geographical, cultural or pandemic-related factors. Given the heterogeneity of our surveyed countries, however, such enquiries are outside the scope of the present study. An interesting topic to explore more with regard to pathogens and social interactions would be to see how disease avoidance affects other, less explored senses such as taste and touch. In fact, a recent study suggests that disease history might be related to affective touch diversity towards a close one [39]. Another important area of future research pertains to how individual differences related to disease avoidance mechanisms translate into behaviours during heightened risk of contamination, such as the COVID-19 pandemics. For example, it is unclear whether the COVID-19 pandemic has increased general levels of xenophobia [40]. However, what the current study shows is that the relationship between levels of BODS and xenophobia is similar to relations observed in an earlier, pre-pandemic study. This could suggest that a salient pathogen threat does not necessarily dramatically affect relations between disgust and attitudes towards fictitious outgroups of varying similarity.

## 5. Conclusion

Individuals more easily disgusted by body odours are also more prone to having negative attitudes towards refugees. As in previous work prior to the COVID-19 pandemic, this relationship was partially explained by the perceived dissimilarity of the outgroup in norms relating to basic practices such as food preparation and hygiene. This main finding was observed in a diverse sample from nine countries across the globe and was similar for attitudes toward refugees from Eastern Africa and Eastern Europe. Our results support the theoretical notion that traditional norms provide protection against pathogens and that outgroups are viewed negatively in part because they are viewed as challenging these norms. Our findings were highly generalizable across countries. The potential sensory components of prejudice should be investigated in more detail to understand the underlying mechanisms, and olfaction seems to be an important factor in disgust-associated behaviours and attitudes.

## Data Availability

Wave one materials and registration: https://osf.io/ycwb3/?view_only=7d411855d55f4ce4a58c36f1dc0c409d. Wave two materials and registration, as well as data from both waves and analysis scripts: https://osf.io/p72m6/?view_only=96f2ea923fd64e6bbf0883a3ec2cde86. Extra tables and information are provided in the electronic supplementary material [41].

## Footnotes

1 Choice of group origin was inspired by Faulkner *et al*. [30].

2 The attitudes towards the COVID-19 pandemic questions had been revised slightly, considering the global development of the pandemic situation that had evolved since the first wave of data collection. Both versions are available on OSF.

3 Specifically, after running Study 1, we realized our first pre-registration was not detailed enough to provide all necessary information needed to recreate (or run) our analysis. Thus, we added (i) details of interaction analysis, which were lacking in the first pre-registration simply due to the fact that we did not realize it requires more than adding an interaction term to the model; (ii) explicit criteria will decide whether data will be analysed country-wise or combined.

4 RMSEA—root mean squared error approximation; SRMR—standardized root mean squared residual; TLI— Tucker–Lewis fit index; CFI—comparative fit index

5 For a brief and informative explanation of Heywood case, we refer the reader to *APA dictionary of psychology* (https://dictionary.apa.org/heywood-case)
